# Influence of inoculum‐to‐substrate ratio on biomethane production via anaerobic digestion of biomass

**DOI:** 10.1111/1758-2229.70009

**Published:** 2024-12-02

**Authors:** Marvin T. Valentin, Daniel Ciolkosz, Andrzej Białowiec

**Affiliations:** ^1^ Department of Applied Bioeconomy Wrocław University of Environmental and Life Sciences Wrocław Poland; ^2^ Department of Agricultural and Biosystems Engineering, College of Engineering Benguet State University Benguet Philippines; ^3^ Department of Agricultural and Biological Engineering Pennsylvania State University, University Park Campus State College Pennsylvania USA

## Abstract

The influence of the inoculum‐to‐substrate ratio (ISR) on anaerobic digestion (AD) of biomass in terms of methane yield and microbial community, was explored in this paper. The level of ISR can affect the AD performance in several ways. At extremely low ISR, volatile fatty acids (VFAs) accumuate, while inhibition occur at higher level of ISR. An ISR ranging from 1.0–2.0 was found optimal resulting in higher methane yield, organic matter removal and VFA degradation. Furthermore, a high ISR (2.0–4.0) is favourable to methanogenesis, while a lower ISR (<1.0) is prone to irreversible acidification. The range of ISR can shift the methanogenic pathway of AD to favour an acetoclastic or hydrogenotrophic response, indicated by the enriched group of microorganisms. The genus *Methanosaeta* (acetoclastic) and *Methanobacterium* (hydrogenotrophic) are the most enriched methanogens across all ISRs, while *Firmicutes*, *Bacteroidetes*, *Proteobacteria* and *Spirochaetae* are dominant in the bacterial community. Additionally, the interplay of substrate biodegradability and ISR potentially affects AD performance. Finally, novel equations are developed and proposed for characterizing the quantity of inoculum and substrate.

## Graphical abstract

The stages of anaerobic digestion of biomass—hydrolysis, acidogenesis, acetogenesis and methanogenesis—are discussed, with an emphasis on the different methanogenic pathways, including hydrogenotrophic and acetoclastic.

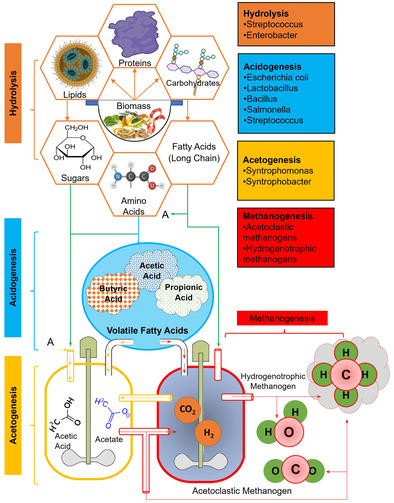

## INTRODUCTION

The degradation of biomass through anaerobic digestion (AD) is one of the most efficient (Codignole Luz et al., 2018; Pham et al., 2015; Zhang et al., 2014) and established strategies to treat biomass and recover energy while producing residual by‐products such as valuable fertilizer (Pan et al., 2019, 2022; Shanmugam et al., 2018). It is a reasonably low‐cost technology (Ambaye et al., 2020, 2021; Liu et al., 2021) that can be applied to a wide range of feedstock, either simple or complex (Achinas et al., 2020; Amha et al., 2018; Papurello et al., 2015; Scholz et al., 2013). AD is carried out through a series of biochemical processes that are mediated by syntrophic microorganisms (Ambaye et al., 2020; Amha et al., 2018; Liu et al., 2021) where the end product of one reaction is the substrate for the next reaction (Nkuna et al., 2022). The growing environment of the microorganisms is important to maintain at optimum conditions, otherwise, inhibition will occur (Fagbohungbe et al., 2017; Ruiz & Flotats, 2014). The degradation of biomass to biomethane involves hydrolysis, acidogenesis, acetogenesis and methanogenesis carried out by corresponding distinct consortia of microbes (Amani et al., 2011; Lee et al., 2012; Liu et al., 2016; Qiu et al., 2019; Song et al., 2018). In the hydrolysis stage, the organic substrates are converted into simple monomers such as lipids, proteins and carbohydrates (Amin et al., 2021) through hydrolytic microbes like *Streptococcus* and *Enterobacter* (Zhao et al., 2021). Acidogenesis is an intermediate breakdown process between hydrolysis and acetogenesis. In this process, soluble monomers such are those complex organic compounds produced during hydrolysis are further degraded by acidogenic bacteria (acid‐forming bacteria) (Amin et al., 2021). Primarily, acidogenesis produces volatile fatty acids (VFAs) including aldehydes, and alcohols (Zhao et al., 2021). During acetogenesis, various organic compounds such as VFAs (acetic acid, propionic acid, valeric acid and butyric acid), and other soluble monomers like long‐chain fatty acids (LFAs) and sugars are converted into acetic acid, CO_2_ and H_2_. Acetate can also be produced at this stage through the reduction of CO_2_ by homoacetogenic bacteria, while the reverse process is accomplished by syntrophic acetate oxidizing bacteria (SAOB) (Amin et al., 2021). The concluding stage in the AD process is methanogenesis where H_2_, CO_2_ and acetate are converted into CH_4_ by methanogens (either hydrogenotrophic or acetoclastic). Throughout these processes, the performance of the AD of biomass can be greatly affected by the inoculum‐to‐substrate ratio (ISR) (Cremonez et al., 2019). The ISR indicates the mass ratio of inoculum (spent digester slurry) to undigested material (digester feedstock or substrate). It is most often used to describe starting conditions of batch‐mode AD experiments, but can also be thought to correlate somewhat to the residence time of continuous feed digesters. Furthermore, the ISR explored in this paper represents instantaneous ISR referring to the ISR of the AD at any time during the operation.

This paper aimed to develop functional equations by integrating the biodegradability of biomass and ISR that can be used to estimate the initial composition of an AD batch reactor experiment in terms of the quantity of the substrate and inoculum. The equations will guide researchers, especially those who are new to the AD process. Furthermore, this concept can be used to better understand and control the AD process for experimental management such as planning for the need for substrate and inoculum addition. The theoretical ISR equation can be used to assess the changes in the concentration of the inoculum and substrate throughout the experiment, which is an indicator of the availability of materials inside the reactor to suggest corrective measures. For instance, a low ISR relative to the ideal level is an indication that there is not enough microbial community to degrade the substrate (Havstad, 2020). We proposed a calculation ISR‐AD‐Tool (Supporting Information Appendix B), which can be useful to simulate the development of ISR during AD batch tests. Finally, the ISR‐AD‐tool was used in this paper for the simulations of ISR development as influenced by the substrate biodegradability.

## INOCULUM AND SUBSTRATE

The AD initially begins when the substrate that is to be digested is mixed with inoculum—fluid from an active digester that is rich in microbes and nutrients. AD requires the appropriate quality and quantity of active inoculum to start digestion (Boulanger et al., 2012; Ma et al., 2019). Previous works have regarded the use of sufficient inoculum as the primary driver to successfully digest complex organic substances (Eskicioglu & Ghorbani, 2011). Aside from influencing the stability of the system, it affects the methanogenetic rate and capacity of the substrate (Li et al., 2022). The inoculum supplies essential microorganisms (Boulanger et al., 2012; Li et al., 2022; Suksong et al., 2019) necessary for the fermentation of primary and intermediate organics (Eskicioglu & Ghorbani, 2011) and is a major factor that affects the dynamic performance of the AD (Rubia et al., 2018). The primary advantage of a suitable ISR are high CH_4_ yield and high chemical oxygen demand (COD) removal (Pagés‐díaz & Huiliñir, 2020).

### ISR range

Varying the ISR shows evidence of its influence on the AD process in terms of resistance to inhibition and accumulation of VFAs, and other pronounced changes especially when the optimum ISR range is exceeded (Pagés‐díaz & Huiliñir, 2020). An ISR of 2.0 is reported to be optimum in most literature based on the VS of the added substrate (Gandhi et al., 2022; Juanga‐Labayen et al., 2021; Lauzurique et al., 2022; Ma et al., 2019; Meng et al., 2018; Moset et al., 2015; Villamil et al., 2017). In some studies, an ISR 1.0 (Villamil et al., 2017), or 0.8–3.0 (Raposo et al., 2009) is acceptable. Higher ISRs are favourable to methanogenesis while irreversible acidification is commonly observed at lower ISR values (Li et al., 2022). AD of lower ISR usually incurs inhibition as manifested by lower pH values of as low as 6.5 which can provoke inhibition (Villamil et al., 2017). ISR below the optimum value favours the production of high ammonia nitrogen and VFA that results in methanogenesis inhibition (Villamil et al., 2017). In terms of the microbial community, an ISR 3.0 favours most bacteria suitable in the AD of food waste (FW) (Li et al., 2022). Improved methane production is associated with bacterial communities with higher diversity (Li et al., 2022; Zheng et al., 2021). Excessive amounts of substrate (lower ISRs) are also related to a longer lag phase and reduction in the CH_4_ yield (Nazaitulshila et al., 2015).

The level of ISR influences the efficiency of the AD process in two ways. At high ISR(low concentration of substrate), there is a risk that microorganisms will reduce their metabolic activity. At low ISR (high concentration of substrate), there is a possibility of overloading the digester, leading to an accumulation of compounds making the system prone to inhibition (Wang et al., 2015). This is in agreement with the findings reported by Nazaitulshila et al. (2015), where methane production stopped at an insufficient amount of microorganisms for biodegradation (Raposo et al., 2009).

ISR is a key parameter that affects the biomethane potential (Dechrugsa et al., 2013). In particular, optimum ISR favours the increase of BMP (Dechrugsa et al., 2013). Alzate et al. (2012) observed higher methane production that ranged from 188 to 395 mL CH_4_/g‐VS_added_ from the digestion of microalgae at ISR 2.0 compared with ISRs 1.0 and 0.33. This aligns with the findings of Raposo et al. (2009) that higher methane yield was obtained at ISR 3.0 compared to ISRs 2.0, 2.5, 0.8 and 5.0 from the digestion of sunflower oil cake in a batch reactor. Contradictory to the findings of Raposo et al. (2006), ISR 1.0 has a higher methane yield of 23 mL CH_4_ g/VS/day compared to 10 mL CH_4_ g/VS/day at ISR 3.0 from the digestion of maize in a mixed batch reactor. The inconsistency of the reports from the literature on the influence of ISR is an indication that other factors should be considered when deciding on the ISR for an experiment such as the type of substrate (Pagés‐díaz & Huiliñir, 2020), source of inoculum (Rubia et al., 2018) and biodegradability.

The variability in the characteristics of the substrate, especially the nutritional composition in terms of C/N ratio content, offers a major challenge during AD. This explains why AD of biomass even under the same ISR level have different yields (Table SA1). ISR directly affects the biodegradability of biomass and the biomethane production (Johnravindar et al., 2023). The level of ISR can also change the microbial structure during AD. Lower ISR triggered the shift of dominance from acetoclastic methanogen (AM) to hydrogenotrophic methanogens (HM), but that did not favour methane production (around 10% reduction) (Xiao et al., 2022). At ISR lower than 1.0, HM and syntrophic bacteria dominated, and methane production decreased by 20% (Xiao et al., 2022). The optimum ISR can correct the nutritional imbalance and serve as a booster to the functional microbial groups. Excess substrates (low ISR) can lead to VFA accumulation and hydrogen buildup (Motte et al., 2013). On the other hand, more inoculation provides greater buffer capacity and more methanogens, but excessive inoculum takes up too much space and decreases the volumetric methane production rate (Xiao et al., 2022).

The organic fraction of the substrate during AD is mostly converted into CH_4_ and CO_2_ especially when following acetoclastic methanogenesis while other fractions are converted into sulphide by sulphate‐reducing bacteria like *Firmicutes* (Amin et al., 2021) and *Desulfotomaculum* sp. (Dechrugsa et al., 2013). This new compound competes with CH_4_ and it is estimated that 5%–15% of the organic matter is removed during the conversion process suggesting that maximum anaerobic degradability is around 85% (Cimon et al., 2020; Rodriguez‐Chiang and Dahl, 2015). Studies concerning the influence of ISRs on the performance of AD and the relative conversion efficiency of methane were analysed and presented in Figure SA1. Good conversion efficiencies from 71% to as high as 85% constituted around 53.5% of the data and these are observed from ISRs at 1.0–2.8. Furthermore, 6.7% of these are greater than the theoretical maximum CH_4_ conversion efficiency (85%) (Cimon et al. 2020; Rodriguez‐Chiang & Dahl, 2015). Though there are some studies on ISR 3.0 and above, they are of limited information. ISRs below 1.0 behaved in two ways. Some (21%) of the ISRs had low conversion efficiencies (4.4%–36.9%) while others were higher than 85%. This does not guarantee the exact relationship of ISRs with the CH_4_ conversion efficiency since other parameters such as the composition of both substrate and inoculum need to be factored in. Furthermore, high degradation efficiencies (>90%) represent 51.8% of reviewed studies, indicated by orange marks, of the observations recorded from biomass of a high biodegradation rate. Only 35.7% (black marks) and 12.5% of the data are from moderate and low biodegradable compounds, respectively (blue marks).

### Impact of substrate quality (source and biodegradability)

The quality of substrate relative to CH_4_ production can be primarily defined by its degradation efficiency (Suksong et al., 2019), expressed as the decomposition constant rate (k). Demichelis et al. (2022) observed a higher biogas yield of 997.81 NL/kg‐VS from the AD of the Organic Fraction of Municipal Solid Waste (OFMSW) with biodegradability of 0.52 day^−1^ as compared to the same substrate with k of 0.47 day^−1^ where biogas yield was only 892.2 NL/kg‐VS. Moset et al. (2015) reported that the optimal ISR for whole‐crop maize is 1.0–1.5 while a wider range of 0.5–2.5 is suitable for a wheat straw substrate. Pagés‐díaz and Huiliñir (2020) observed better methane yield ranging from 52 to 83 NmLCH_4_/g tCOD_added_ at ISR 2.0 from the AD of liquid fractions of co‐hydrothermally treated mixed sewage sludge and OFMSW. Other works reported good results at an ISR 1.0 (Pagés‐díaz & Huiliñir, 2020; Raposo et al., 2009) indicating that the optimum ratio depends greatly on the substrate's biodegradability (Lauzurique et al., 2022). Substrate composition can be toxic to microorganisms especially at excessively low ISRs as observed from the BMP of maize that was inhibited (14.4 CH_4_ kg/VS) as compared to wheat straw where it was not affected (287.3 CH_4_ kg/VS) at the same ISR 0.25 (Moset et al., 2015). In addition, initial COD concentration can also impact biodegradability across ISRs. Inoculum having an initial COD concentration of 25 g‐COD/L at ISR 2.0, compared to lower ISRs, shows strong resistance to methanogenesis inhibition as indicated by higher methane production (177 mL‐CH_4_ STP/g‐COD_added_) (Villamil et al., 2017).

These findings indicate that biodegradability has to be considered when the ISR is selected in the AD process. In the case of highly biodegradable substrates, ISR should be higher to avoid quick acidification due to the rapid production of VFA. Whereas, less biodegradable compounds (low k an ISR with lower values may be chosen. However, up to date, there are no specified threshold values of ISRs that are associated with specific, k values. A method for selecting an appropriate ISR for varying biodegradability values would be a valuable tool for AD researchers and system operators.

## INOCULUM‐TO‐SUBSTRATE RATIO CALCULATION

The calculation of ISR has been done using several different approaches, making it challenging to prepare comparative remarks among similar studies (Lauzurique et al., 2022; Pagés‐díaz & Huiliñir, 2020). Some of the bases of calculation include the total COD of the substrate (Pagés‐díaz & Huiliñir, 2020), mass in grams of soluble COD (Lauzurique et al., 2022) and mass in grams of VS (g‐VS) of the substrate (Moset et al., 2015), summarized in Table 1. In these equations, the biodegradability of substrates and inoculum is not taken into account. Thus, if these equations are used, two experiments could have the same ISR but still have different methane yields. Likewise, when setting up a specific initial volume of the experiment, these equations cannot be directly applied.

**TABLE 1:** Different equations used in the literature for the calculation of inoculum‐to‐substrate ratio (ISR).

Equations	References
$\mathrm{SIR}=\frac{g{\mathrm{VS}}_{\text{subs}}}{g{\mathrm{VS}}_{\text{inoc}}}$	(Moset et al. 2015)
$\mathrm{ISR}=\frac{V_{\text{inoc}}{\mathrm{VS}}_{\text{inoc}}}{V_{\text{subs}}\;{\mathrm{COD}}_{\text{subs}}}$	(Ambaye et al., 2020)
$\mathrm{ISR}=\frac{\mathrm{gVS}\;\text{Inoc}}{\mathrm{gVS}\;\text{Subs}}$	(Gandhi et al., 2022)
$\mathrm{ISR}=\frac{\text{Inoc concentration}}{\text{Subs concentration}}$ $\mathrm{ISR}=\frac{g{\mathrm{VS}}_{\text{inoc}}/L\;}{g{\mathrm{VS}}_{\text{subs}}/L}$	(Raposo et al., 2009)
$\mathrm{ISR}=\frac{\frac{\text{gCOD}}{L}\;\text{inoc}}{\frac{\text{gCOD}}{L}\;\text{subs}}$	(Villamil et al., 2017)
$S/I=\frac{g\;\text{soluble}\;\mathrm{COD}}{\text{gVSS}}$	(Lauzurique et al., 2022)

*Note*: Subs, Inoc, gVS and gVSS mean substrate, inoculum, mass in gram of volatile solids, and mass in gram of volatile suspended solids.

### Proposed ISR calculation—ISR‐AD‐tool

In an AD experiment, the quantity of the mixture is often related to the working volume of the available reactor in the laboratory. The working volume of the reactor is comprised of the individual volume of the inoculum, substrate and nutrient solution excluding headspace (Equation 1). The nutrient solution depends on the requirement of the experiment. It can be disregarded if the substrate being treated does not require nutrients leaving the total working volume with the substrate and inoculum only.

(1) $$V_{w}=V_{\text{subs}}+V_{\text{inoc}}+V_{\mathrm{ns}}$$

where $V_{w}$ is the reactive or working volume of the reactor to accommodate the volume of substrate ($V_{\text{subs}}$), the volume of inoculum ($V_{\text{inoc}}$), the volume of the nutrient solution ($V_{\mathrm{ns}}$) and additional materials as needed in the experiment. The working volume is usually a certain percentage of the total reactor volume. Most literature used 80% as a working volume (Namal, 2020; Rodriguez‐Chiang & Dahl, 2015; Wang et al., 2015). The remaining 20% is dedicated for the headspace. The degradation of the volatile matter content of the biomass can be described by an ordinary differential equation following physical law of time rate of change which assumes that the change in VS is proportional to time (Equation 2).

(2) $$\frac{\mathrm{dVS}}{\mathrm{VS}}\propto -\mathrm{kdt}$$

where VS denotes the amount of volatile solids, and k (time^−1^) is the proportionality constant, more appropriately known as biodegradability, assigned with a minus sign indicating mass loss due to degradation. Applying integration to Equation 2 and implementing limits from time zero to time t results in $\ln\left[{\mathrm{VS}}_{t}\right]=-\mathrm{kt}+c$, where ${\mathrm{VS}}_{t}$ is the volatile solid at any time t simplified into its exponential form (Equation 3).

(3) $${\mathrm{VS}}_{t}={\mathrm{ce}}^{-\mathrm{kt}}$$

In the initial value condition, at time zero (t=0), the amount of volatile solid present in the reactor is simply its initial quantity (${\mathrm{VS}}_{t=0}\to {\mathrm{VS}}_{\text{intl}}$), say, initial volatile solid (${\mathrm{VS}}_{\text{intl}}$). Applying this condition to Equation (3) gives the value for the constant c ($c={\mathrm{VS}}_{\text{intl}}$) resulting in a particular solution (Equation 4).

(4) $${\mathrm{VS}}_{t-s}=\left({\mathrm{VS}}_{\text{intl}}\right)\;e^{-k_{s}t}$$

where ${\mathrm{VS}}_{t-s}$ is the VS of the substrate at any time and $k_{s}$ is the biodegradability constant of the substrate. In the case of inoculum, microbial biomass growth can occur due to the decomposition of volatile solids from the substrate. Considering that microbial biomass growth follows a first‐order character, the equation describing the accumulation of microorganisms can have the form (Equation 5).

(5) $${\mathrm{VS}}_{t-i}={\mathrm{VS}}_{\text{imax}}\left(1-e^{-k_{i}t}\right)$$

where ${\mathrm{VS}}_{t-i}$ is the VS of the inoculum at any time, ${\mathrm{VS}}_{\text{imax}}$ is the asymptotic value of the maximum biomass growth and $k_{i}$ is the biodegradability constant of the inoculum. In the preparation of the mixture of substrate and inoculum, their respective quantities are usually based on the volatile solid content of the materials (Equation 6) similar to the equations indicated in Table 1.

(6) $$\mathrm{ISR}=\frac{\text{Mass in grams of}\;{\mathrm{VS}}_{\text{inoc}}}{\text{Mass in grams of}\;{\mathrm{VS}}_{\text{subs}}}$$

Considering the change in VS of inoculum and substrate over time, Equations (4) and (5) can be integrated and substituted into Equation (6) to give Equation (7). In some works, ISR is expressed in terms of the COD or even in terms of the auto‐catalytic process relating to the existing microbial cells in the inoculum. The inoculum originally contains microbial cells which grow exponentially as it consumes the substrate during AD. With this, the ability of the substrate to degrade (biodegradability) and be made available for microorganisms also affects the growth of the cells.

(7) $$\mathrm{ISR}=\frac{{\mathrm{VS}}_{\text{imax}}\left(1-e^{-k_{i}t}\right)\;}{{\mathrm{VS}}_{\text{subs}}\;e^{-k_{s}t}}$$

The entire numerator of Equation (7), ${\mathrm{VS}}_{\text{imax}}\left(1-e^{-k_{i}t}\right)$ represents the VS of the inoculum (${\mathrm{VS}}_{\text{inoc}}$) and it increases with time. This is for the reason that microorganisms consume the substrate by fermentation. With this, the microorganisms in the inoculum grow leading to an increase in its volatile solid content due to the principle of biomass growth. The biomass growth expressed as g‐VS added cannot be higher than the VS consumed from the substrate. Therefore, the VS of inoculum can be expressed in Equation (8).

(8) $${\mathrm{VS}}_{\text{inoc}}=\left({\mathrm{VS}}_{\text{subs}\left(\text{intl}\right)}-{\mathrm{VS}}_{\text{subs}\left(\mathrm{eff}\right)}\right)\left(1-e^{-k_{i}t}\right)+{\mathrm{VS}}_{\text{inoc}\left(\text{intl}\right)}$$

(9) $${\mathrm{VS}}_{\text{inoc}}={\mathrm{VS}}_{u}\left(1-e^{-k_{i}t}\right)+{\mathrm{VS}}_{\text{inoc}\left(\text{intl}\right)}$$

In Equation (8), the difference in the initial VS of the substrate (${\mathrm{VS}}_{\text{subs}\left(\text{intl}\right)}$) and final (effluent) VS of the substrate (${\mathrm{VS}}_{\text{subs}\left(\mathrm{eff}\right)}$) is the amount of VS solids available for the microorganisms. In other studies, this is referred to as the ultimate VS degraded at the time, t (Kavitha et al., 2016). For simplicity, this difference is renamed as, ${\mathrm{VS}}_{u}$ (Equation 9). Biomass growth from this scenario will be in the order $1-e^{-k_{i}t}$. Adding this to the initial VS of the inoculum (${\mathrm{VS}}_{\text{inoc}\left(\text{intl}\right)}$) can give the VS of the inoculum at any time. The final VS of the substrate can be calculated using Equation (4) considering the half‐life principle ($t_{1/2}=\ln2/k_{s}$). With this, Equation (9) can be integrated into Equation (4) to give Equation (10).

(10) $$\mathrm{ISR}=\frac{{\mathrm{VS}}_{u}\left(1-e^{-k_{i}t}\right)+{\mathrm{VS}}_{\text{inoc}\left(\text{intl}\right)}\;}{{\mathrm{VS}}_{\text{subs}}\;e^{-k_{s}t}}$$

The mixture is more convenient to prepare in terms of the respective initial masses of the materials. As such, Equation (10) is expressed in terms of mass (Equation 11) where, ${\mathrm{FM}}_{i}$, refers to the fresh mass of the inoculum, ${\mathrm{FM}}_{\text{subs}}$, refers to the fresh mass of the substrate, ${\mathrm{DM}}_{\text{inoc}}$ and ${\mathrm{VS}}_{\text{inoc}}$, are the percent dry mass and percent volatile solid of the inoculum in decimal, respectively, while ${\mathrm{DM}}_{\text{subs}}$ and ${\mathrm{VS}}_{\text{subs}}$ represent the percent dry mass and percent volatile solid of the substrate in decimal, respectively.

(11) $$\mathrm{ISR}=\frac{{\mathrm{VS}}_{u}\;\left(1-e^{-k_{i}t}\right)+{\mathrm{FM}}_{\text{inoc}}{\mathrm{DM}}_{\text{inoc}}V_{\text{inoc}}\;}{{\mathrm{FM}}_{\text{subs}}{\mathrm{DM}}_{\text{subs}}{\mathrm{VS}}_{\text{subs}}\;e^{-k_{s}t}}$$

Furthermore, the ISR can be expressed in terms of the corresponding volume of inoculum and substrate (Equation 12), where $V_{\text{inoc}}$ and $V_{\text{subs}}$ are the volumes of inoculum and substrate, respectively, and $\rho_{\text{inoc}}$ and $\rho_{\text{subs}}$ are the respective bulk densities of the inoculum and substrate, respectivel. This would be convenient to use especially if the substrate is in liquid form. However, in most cases, the substrate comes in solid form while the inoculum is liquid. With this, the denominator of Equation (11) is written in terms of fresh mass.

(12) $$\mathrm{ISR}=\frac{{\mathrm{VS}}_{u}\left(1-e^{-k_{i}t}\right)+\rho_{\text{inoc}}\;V_{\text{inoc}}{\mathrm{DM}}_{\text{inoc}}{\mathrm{VS}}_{\text{inoc}}\;}{\rho_{\text{subs}}\;V_{\text{subs}}{\mathrm{DM}}_{\text{subs}}{\mathrm{VS}}_{\text{subs}}\;e^{-k_{s}t}}$$

### Inoculum concentration

**FIGURE 1:**
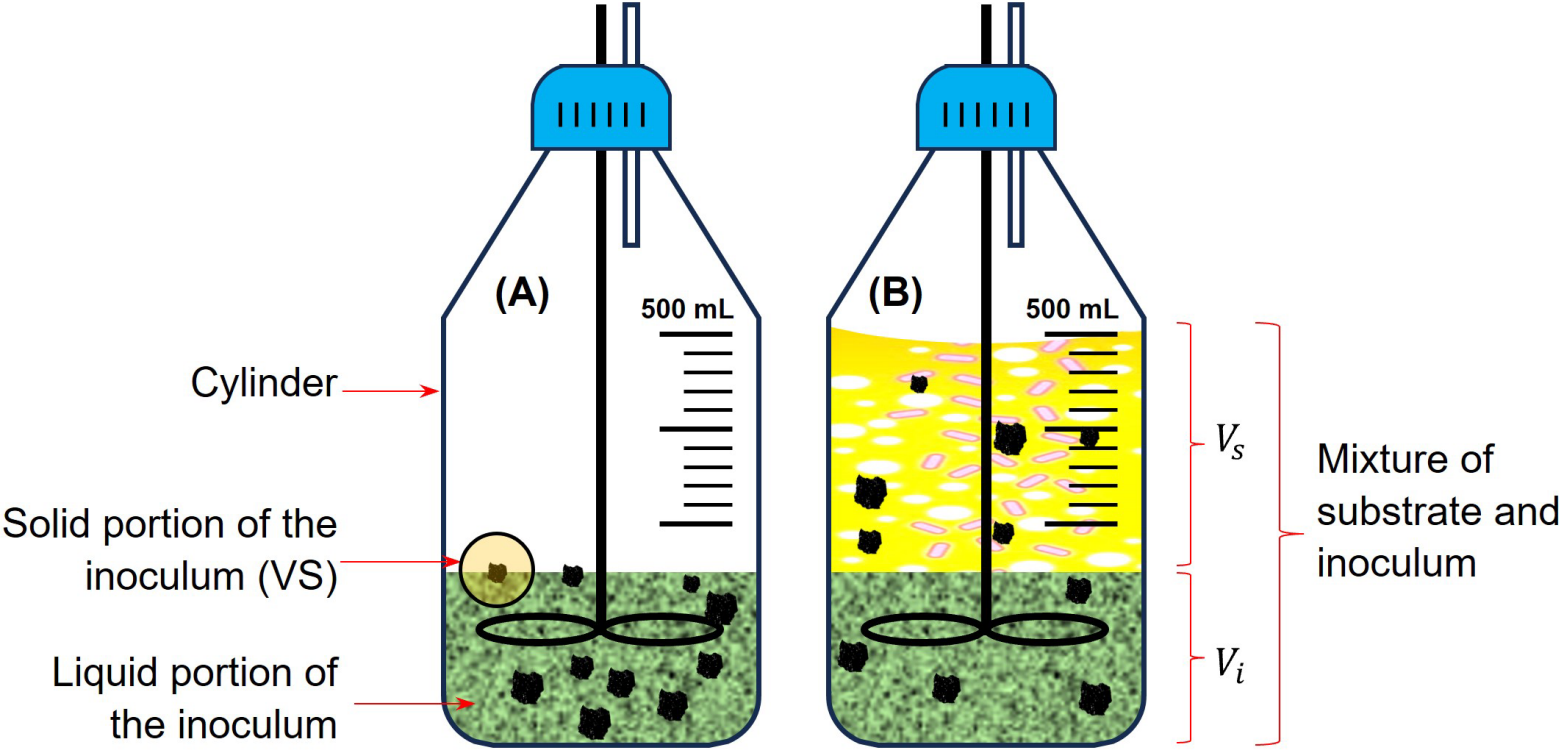
Sludge concentration relative to the original quantity of the inoculum and to the total reactive volume occupied by substrate and inoculum.

The inoculum concentration (IC) or sludge concentration is an important characteristics of the inoculum that affects methane production and is independent of the effects of ISR (Villamil et al., 2017). IC relates to the concentration of the volatile solid or COD of the inoculum to the total reactive volume. This dictates the quantity of dilution in the reactor. IC varies in the literature such as 10 g‐VS/L (Ambaye et al., 2020; Dechrugsa et al., 2013), 8 g dry weight DW/L (Cai et al., 2016), or even higher. Villamil et al. (2017) reported that IC at 25 g COD/L mixed at ISR 2 had the highest methane yield of 177 mLCH_4_ STP/g‐COD_added_ in the AD of the liquid fraction from hydrothermal carbonization of dehydrated sewage sludge. The IC in the reactor is given in Equation (13) which describes the concentration of the inoculum relative to the total working volume. Figure 1A illustrates the volatile solids concentration of the inoculum relative to its initial volume. The volatile solids are represented by the black particles with definite mass, thus the IC is 10 particles/$V_{i}$ (g/cm^3^). When the inoculum is mixed, for digestion, with other substances such as substrate, water and/or nutrient solution constituting the total working volume then the new IC relative to the total working volume is 10 particles/$V_{w}$, which is expected to decrease because the new volume has increased (Figure 1B).

(13) $$\mathrm{IC}=\frac{{\mathrm{FM}}_{\text{inoc}}{\mathrm{DM}}_{\text{inoc}}{\mathrm{VS}}_{\text{inoc}}}{V_{w}}$$

Hence, the volume of inoculum when IC is integrated in the reactor is given in Equation (14). These two equations can be expressed both in volume or mass to meet the preference of the user.

(14) $$V_{\text{inoc}}=\frac{\mathrm{IC}V_{w}}{\rho_{\text{inoc}}V_{\text{inoc}}{\mathrm{DM}}_{\text{inoc}}{\mathrm{VS}}_{\text{inoc}}}$$

### Changes in ISR during batch experiments

In the preparation of the AD, the mixture is set to the optimum ISR value, such as 2.0. As the digestion progresses, the volatile solid of the substrate is digested by bacteria for biogas production, while the inoculum continues to accumulate volatile solids. The simulation of the influence of the ${\mathrm{VS}}_{i}$ and ${\mathrm{VS}}_{s}$ on ISR throughout the AD operation has been proposed as described in the Supporting Information Appendix B (Table SB1), with the application of the proposed ISR‐AD tool. The changes in the ISR was based on a 20‐day expiremental period with the initial physical properties of inoculum and substrates determined experimentally at the laboratory. Initially, the substrate has a volatile solid of 2.61 g‐VS while the inoculum has 5.22 g‐VS. This mixture gives an initial ISR of 2.0 at the start of the experiment. At the end of the 20th day, the substrate is left with 0.237 g‐VS where 2.373 g‐VS is degraded for biogas conversion. Conversely, the inoculum grows to 5.446 g‐VS at the end of the experiment.

**FIGURE 2:**
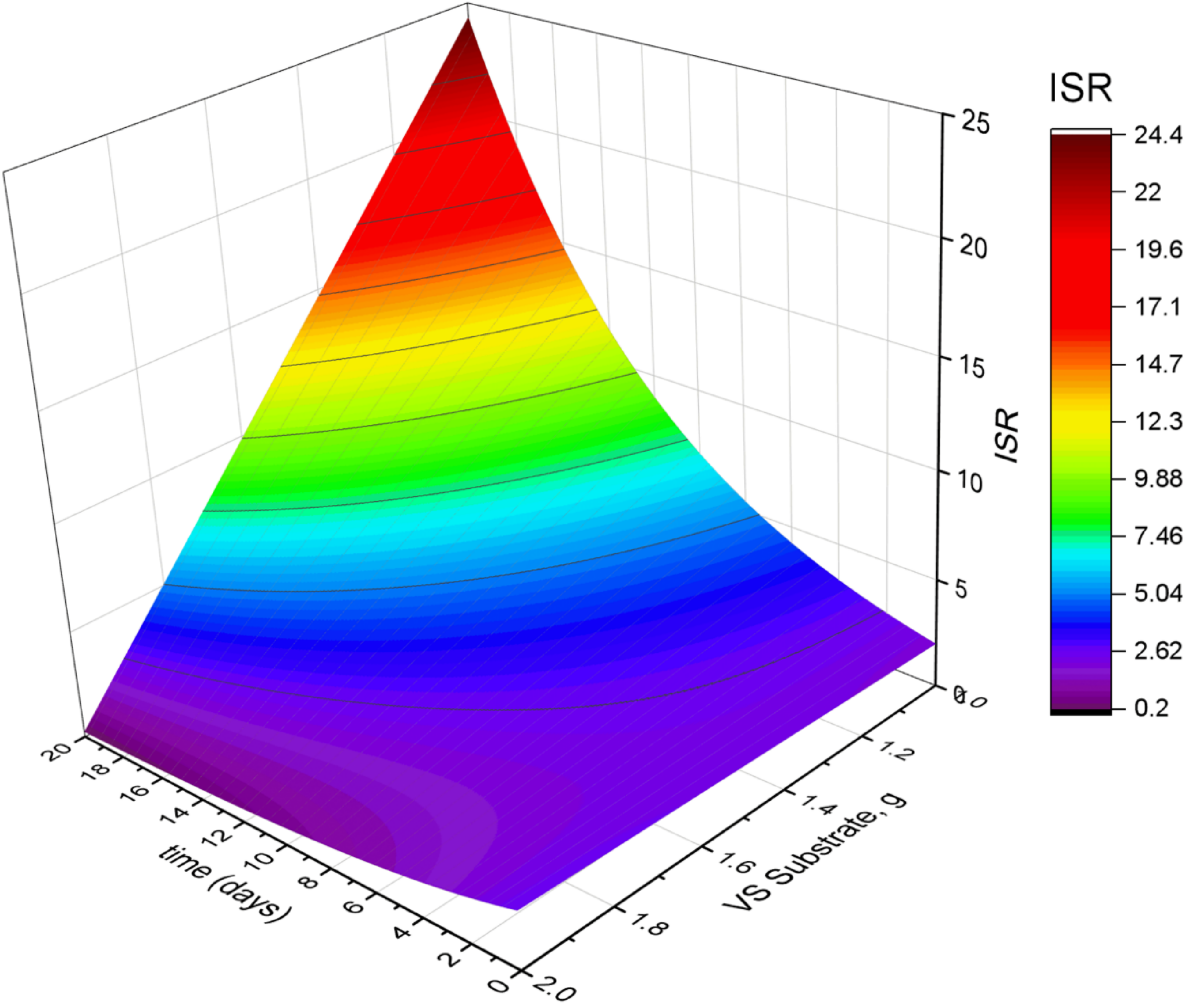
The change in the inoculum‐to‐substrate ratio (ISR) during the anaerobic digestion of biomass.

Figure 2 shows the exponential change of the ISR over time modelled using Equation (10). The substrate and inoculum has a biodegradability (k) of 0.005 and 0.12 day^−1^, respectively, and initial volatile solids of ${\mathrm{VS}}_{s}=2.61\;g$ and ${\mathrm{VS}}_{i}=5.22\;g$ for a 20‐day AD time (Figure 4). The ISR increased throughout the process which indicates the decrease of the substrates (Figure SB1). The amount of volatile solids in the inoculum increases exponentially while decreasing exponentially for the substrates (Figure SB2).

**FIGURE 3:**
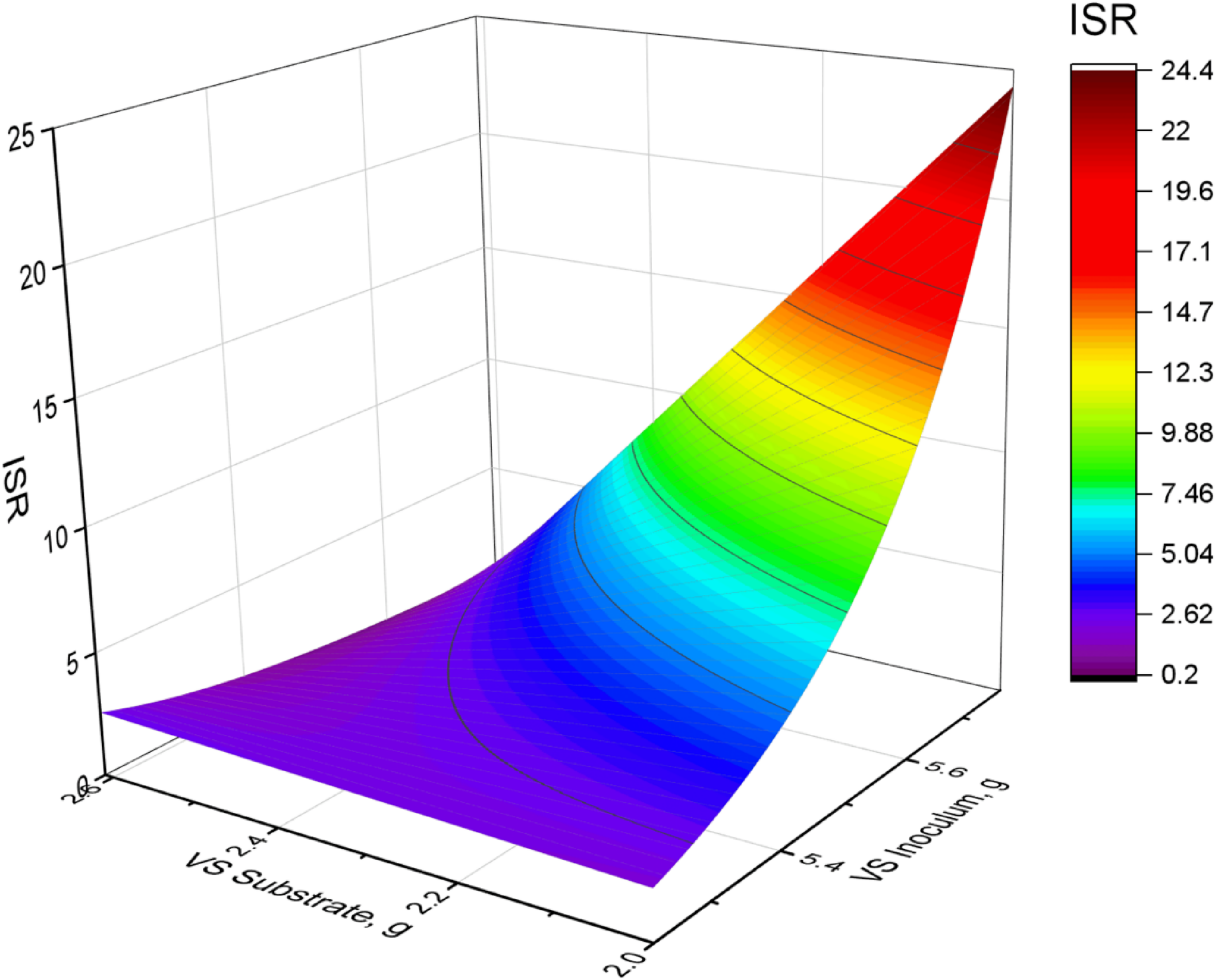
The change in volatile solids of the substrate and inoculum as affected by the change in the ISR of the mixture in the AD.

**FIGURE 4:**
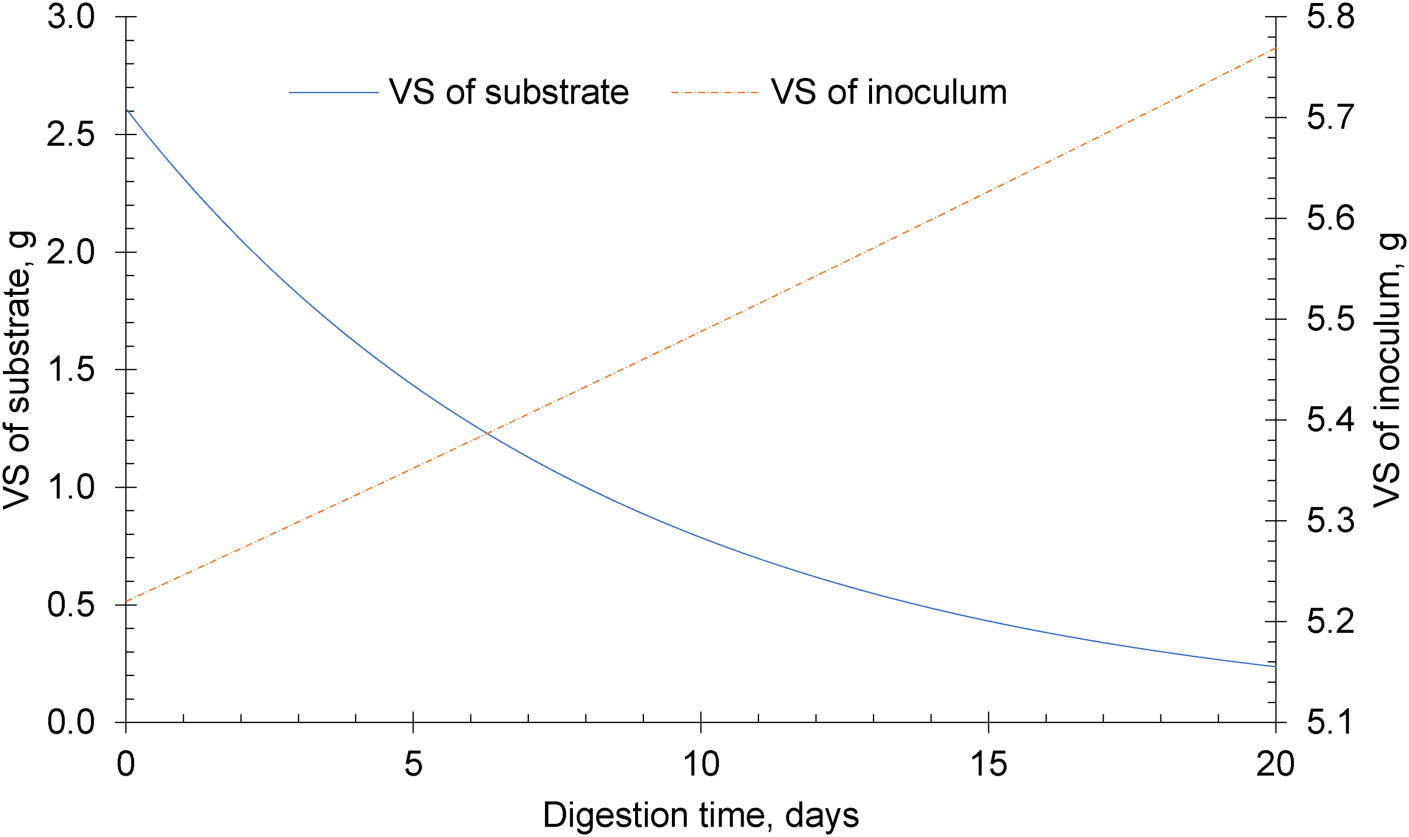
The change in the volatile solids of the substrate and the inoculum with corresponding biodegradability of $k_{i}=0.005$ and $k_{s}=0.12$ throughout a 20‐day digestion time.

The change in the volatile solids of the substrate and inoculum at different ISRs throughout the digestion time is shown in Figures 3 and 4. This illustrates the rate of microbial activity in the reactor with the degradation of the substrate. The exponential decrease of the substrate further indicates that microorganisms in the inoculum exhibit exponential growth causing an exponential degradation of the organic matter. The proposed ISR‐AD‐Tool may be used for modelling ISR development in batch tests with the application of different values of ${\mathrm{VS}}_{i}$, ${\mathrm{VS}}_{s}$, the density of substrate and inoculum, the volume of the reactor, and k values of the degradation and growth rate.

**FIGURE 5:**
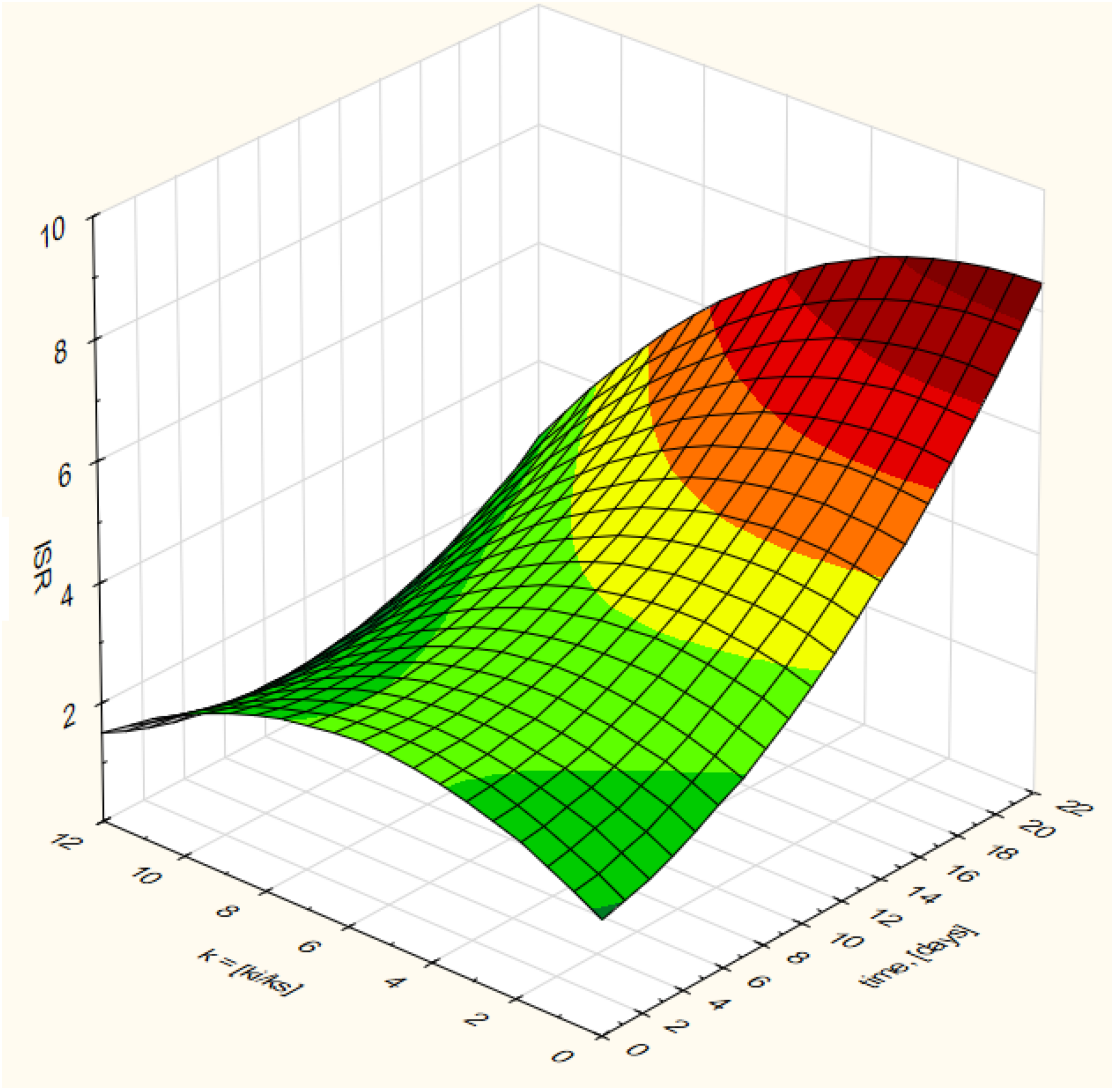
The simulated change in the ISR at different values of biodegradability of inoculum and substrate ($k_{i}/k_{s}$) for 20 days AD batch test.

The different levels of $k_{i}$ ranging from 0.1 to 0.0001 and $k_{s}$ ranging from 0.1 to 0.009 (Table SB2) were used to assess the change in ISR over a 20‐day AD batch experiment. The simulated values of the ISR and the corresponding volatile solids of the substrate and inoculum and their changes over the AD time under different variants are shown in Table SB3. The summary of the changes in ISR throughout the AD process as influenced by the biodegradability of the substrate and inoculum is summarized in Table SB4. The biodegradability was expressed in terms of $k_{i}/k_{s}$ (Table SB5). A 3D surface plot resulting from a quadratic model simulation in Statistica and Origin Pro is shown in Figure 5. In the simulation, it shows that slight changes in ISR from its initial value of 2.0 were observed from the biodegradability ratio of $k_{i}/k_{s}$ between 10 and 12 (hardly biodegradable substances). Consequently, the ISR could rise to 8.0 or more at the end of the AD period for $k_{i}/k_{s}$ less than 1.0 (highly biodegradable substances). The simulation provided a quadratic model shown in Equation (15) that describes the 3D surface plot, which relates the change in the ISR as affected by the substrate biodegradability and the process time. The model has a coefficient of determination ($R^{2}$) of 78.5%, and root mean square error (RMSE) of 3.98.

(15) $$\mathrm{ISR}=\begin{array}{l} 1.940+0.088t+0.304\left(\frac{k_{i}}{k_{s}}\right)-0.009t^{2}\\ -\;0.0216t\left(\frac{k_{i}}{k_{s}}\right)-0.025{\left(\frac{k_{i}}{k_{s}}\right)}^{2} \end{array}$$

## MICROBIAL COMMUNITY

The microbial community of AD reported in the literature under various ISRs is summarized and discussed in this section. In particular, the effect of ISR on the microbial population both for the bacteria and archaea is taken into consideration.

### Bacterial community

**FIGURE 6:**
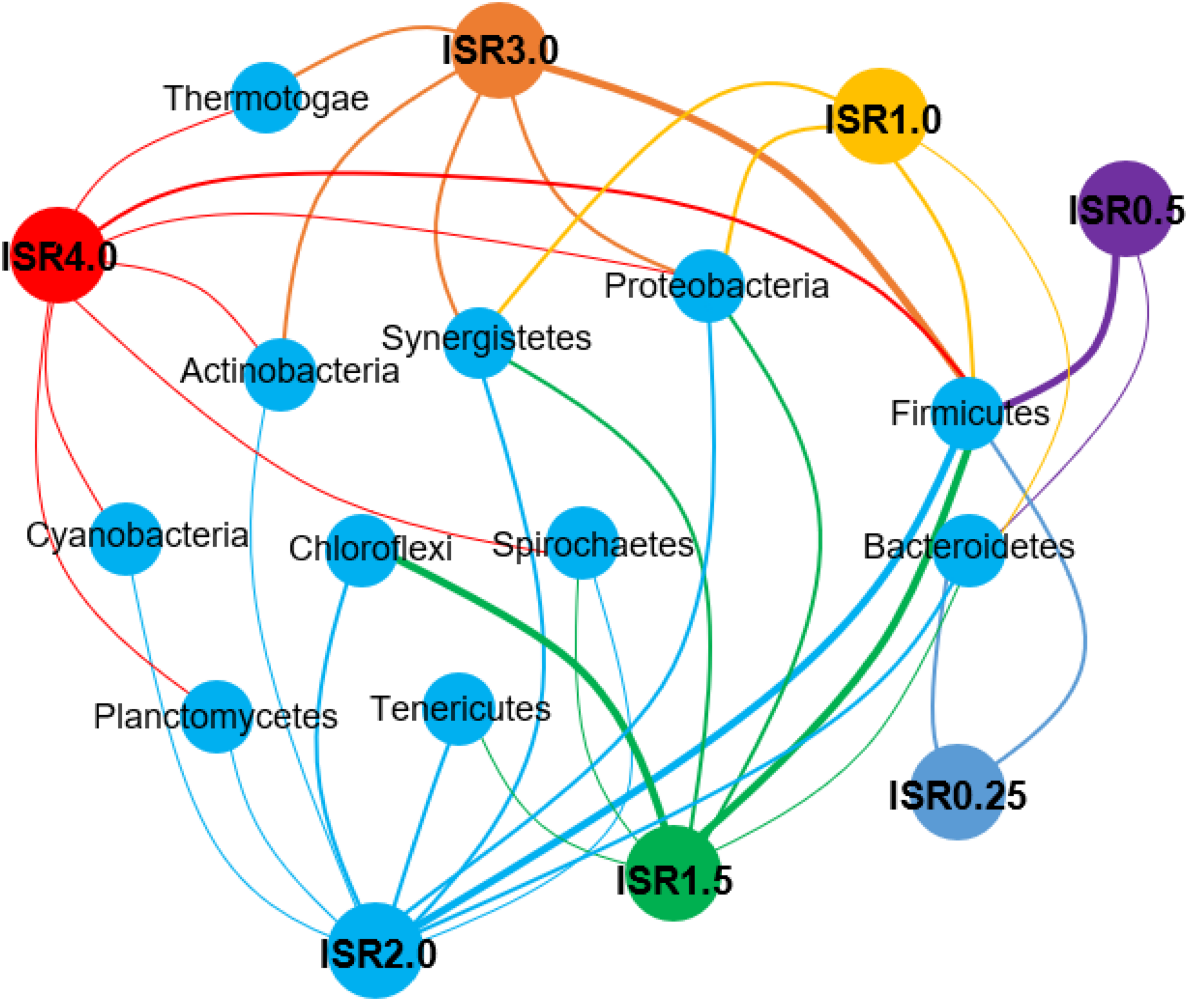
Common acetogens present in anaerobic digesters. The size of the circles indicates the relative abundance of each acetogen, and the thickness of the lines represents the relative strength of the link.

The major phyla present in inoculum used in previous works are likely universal in AD with lignocellulosic feedstock containing an abundance of *Firmicutes* (Ma et al., 2019), *Bacteroidetes*, *Proteobacteria*, *Spirochaetae*, *Tenericutes*, *Synergistetes*, *Chloroflexi* and *Planctomycetes* (Li et al., 2016; Zhao et al., 2016; Zheng et al., 2015) (Figure 6). The significance of bacterial communities indicates that higher diversity contributes to more efficient methane production (Zheng et al., 2021). The bacterial community is more diversified at higher ISRs (Li et al., 2022). For instance, at an ISR of 1.5, the operational taxonomic units (OTUs) for bacteria were 7668 while they were only 540 at 0.25 with the same substrate (Ma et al., 2019). Ma et al. (2019) observed that the population of bacteria in ISR 1.5 reached 7.6 × 1010 copies/g VS, compared to 2.4 × 10^8^ copies/g‐VS at 0.25. Another indicator is the Shannon index in which ISR 3.0 was the highest (5.6) among other ISRs indicating a more diversified bacterial community (Li et al., 2022). In terms of sobs index, ISRs 2, 3 and 4 had respective indexes of 674.50, 685.75 and 679.00 compared to a lower value of 577.17 at 0.25 indicating that higher ISR cultivated the growth and reproduction of bacteria (Li et al., 2022).

In a solid‐state AD at ISR of 1.5, *Firmicutes* was relatively abundant compared with ISR 0.25 with higher VFA concentrations (25.4 g/L) and lower pH (5.9) in digesters where *Bacteroidetes* was dominant (Ma et al., 2019) suggesting that *Firmicutes* can be suppressed at such conditions while *Bacteroidetes* is more resistant. Li et al. (2022) reported that in the AD of FW, *Firmicutes* at ISR 0.25 was the most abundant (65.11%) even in the acidic environment. Hydrolytic and acidogenic bacteria that hydrolyze and transform organic matter (Yang et al., 2021) such as *Bacteroidetes* and *Synergistetes*, became more abundant at the increased quantity of inoculum with maximum abundance at ISR of 3.0 leading to HM pathway (Li et al., 2022). A digester with rape straw and dairy manure showed a greater abundance of *Firmicutes* and *Bacteroidetes* optimal ISR resulting in greater degradation of the cellulose and hemicellulose components and higher production of VFAs (Ma et al., 2019). *Firmicutes* and *Bacteroidetes* hydrolyze and ferment fibre into organic acids, and they are related to VFA production (Li et al., 2016; Ma et al., 2019; Zheng et al., 2015).

### Archaeal community

**FIGURE 7:**
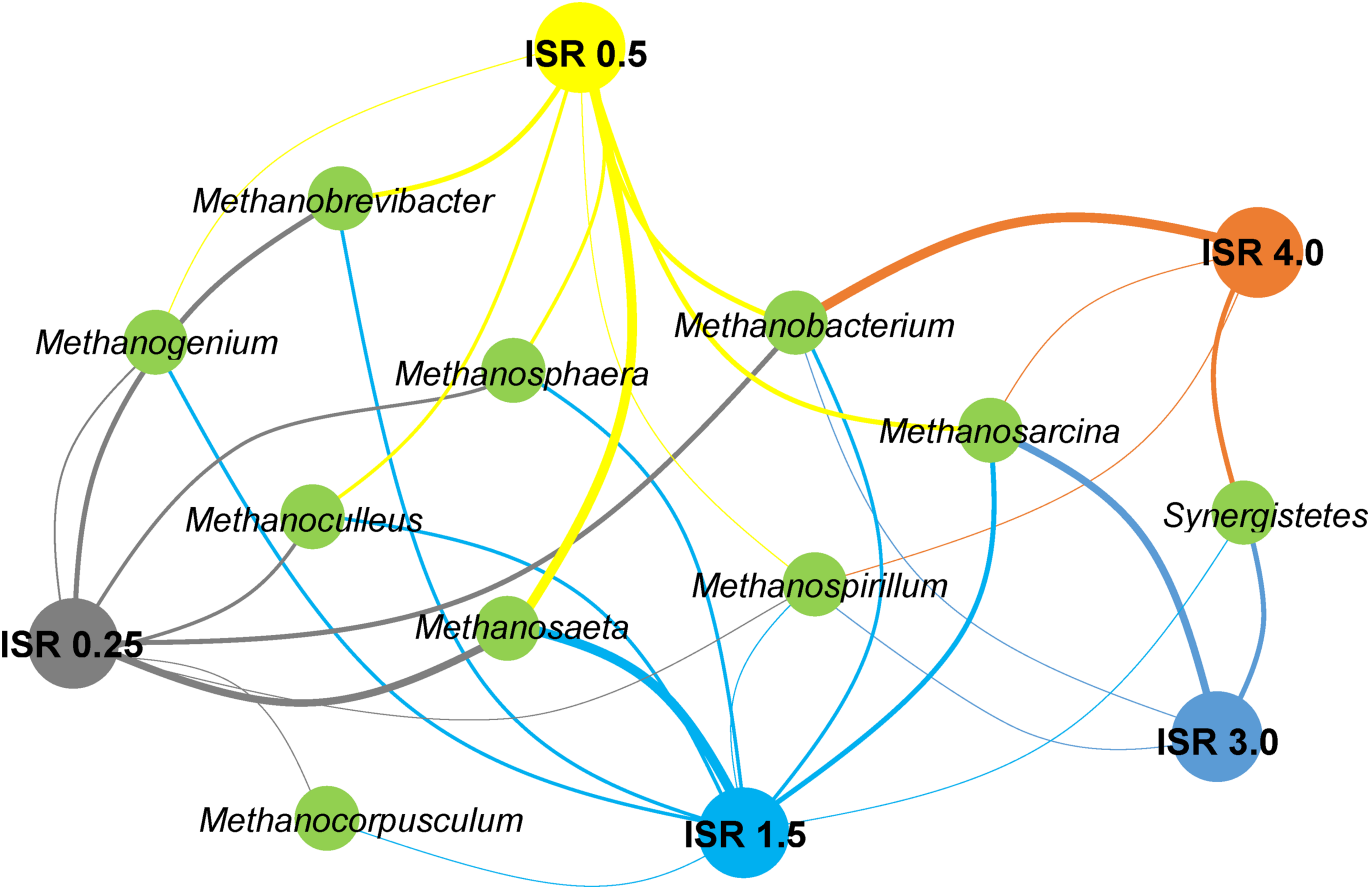
Network map of a relatively abundant archaeal community (methanogens) as influenced by the ISRs. The thicker the lines indicate a stronger link.

The pre‐dominant archaea common to inoculum were *Methanosaeta*, *Methanosarcina*, *Methanobacterium*, *Methanobrevibacter*, Methanoculleus and *Methanosphaera* (Ma et al., 2019). Some of these Euryarchaeota including *Methanospirillum* were also recorded in the AD of FW across different ISRs (Li et al., 2022). Luo et al. (2015) attested that *Methanobacterium*, *Methanosaeta* and *Methanosarcina* were the most abundant methanogens constituting over 90% of the archaeal community in the AD of glucose. Furthermore, digesters were classified by Ma et al. (2019) as either healthy (ISRs, 1.5 and 0.5) or sour (ISR, 0.25) and the relative abundance of these archaea differed between the group (Ma et al., 2019). ‘Healthy’ digesters steadily produced methane even after some time and are found to exhibit similar microbial profiles with the inoculum (Li et al., 2016). A network map of the relatively abundant methanogens in digesters affected by different levels of ISRs (Li et al., 2022; Ma et al., 2019) is presented in Figure 7. The lines between the ISRs and the methanogens represent linkage strength as indicated by the relative abundance of the methanogens. Ma et al. (2019) reported that genus *Methanosaeta* was most abundant in the archaeal community from the anaerobic co‐digestion of rape straw and dairy manure among ISRs of 1.5 (55.6%), 0.5 (3.2%) and 0.25 (39.8%) followed by *Methanoculleus*, *Methanobrevibacter* and *Methanosphaera*. A different observation was reported by Li et al. (2022) wherein *Methanobacterium* was the most abundant in the AD of FW particularly at ISR 3.0 and 4.0 with the corresponding relative abundance of 75.11% and 60.53% indicating that hydrogenotrophic methanogenesis was the primary pathway in the digestion. The genera *Methanobrevibacter*, *Methanosphaera* and *Methanogenium* increased sharply in the acidic digester and decreased at ISRs 1.5 and 0.5 (Ma et al., 2019). These genera belonging to the *Methanobacteriaceae* family of the *Methanobacteriales* order are more abundant in environments with high total acid concentrations and low pH (Blume et al., 2010).

Archaeal community in the AD of FW is reported to have more diversity at higher ISRs (Li et al., 2022). At ISR 3.0, *Synergistetes* was abundant which resulted in higher methane production as compared to ISR 0.25 (Li et al., 2022). *Synergistetes* can stimulate methane production through interspecies electron transfer (IET) and can establish syntropy with hydrogenotrophic methanogens (*Methanobacterium* and *Methanospirillum*) (Yang et al., 2021). The decrease in ISR was observed to shift the hydrogenotrophic methanogenic pathway from the acetoclastic pathway indicated by the relative abundance of methanogens such as *Methanolinea* and *Methanosarcinales* (Xiao et al., 2022). In the AD of food waste, the HM *Methanolinea* of *Methanomicrobia* had a relative abundance of 2%, 9% and 11% at ISRs of 3.3, 2.0 and 1.0, respectively, while 66% at ISR 0.5 (Xiao et al., 2022) suggesting that acetoclastic methanogenesis pathways are favourable at higher ISRs. However, still, the same study observed that methane production was unfavourable in both scenarios because at ISR 1.0 where AM *Methanothrix* belonging to *Methanosarcinales* had a relative abundance of 71%, and yet the methane production was reported to be lowest among other ISRs (Xiao et al., 2022). This suggests that highly abundant *Methanosarcinales* decarboxylated the CH_3_COO^−^ to CO_2_ instead of CH_4_. *Methanosaeta* and *Methanosarcina* are important in carrying out acetoclastic methanogenesis (Ma et al., 2019). *Methanosaeta* can help improve methane production even at high acetic acid concentrations (Zhao et al., 2016). At lower ISR particularly in the case of glucose as substrate, the abundance of HM was replaced by acetoclastic *Methanosaeta* (Luo et al., 2015). The methane production at ISR 1.0, where HM *Methanolinea* belonging to *Methanomicrobiales*, was 11% higher than other ISRs (3.3 and 2.0) which had the lowest methane production.

## RECOMMENDATIONS

The significance of ISR as a major parameter in the AD process is evident from the review of related studies available in the literature. ISR can significantly influence the performance of AD in several ways including resistance to inhibition and accumulation of VFAs (VFA). ISR can influence methanogenic pathways of the AD system whether hydrogenotrophic or acetoclastic methanogenesis predominates. Optimum ISR is established between 1.0 and 2.0 in most literature. In addition, the type and source of inoculum and substrate are also influential in AD performance which could be defined by their respective biodegradability. With this, a useful and novel equation has been developed in this work, and the novel ISR‐AD‐Tool has been proposed to readily calculate the quantity of inoculum and substrate during batch AD experiments at the laboratory. Overall ISR at 2.0 has higher CH_4_ compared to extremely low or high ISR where methane production is inhibited. The abundance, diversity and uniformity of microorganisms, in terms of the microbial community, is higher at ISR 2.0 and beyond compared to lower ISR. The dominant bacteria recorded for ISR 2.0 were *Firmicutes*, *Chloroflexi*, *Proteobacteria* and *Bacteroidetes*. Though there are no distinct reports regarding the methanogens for ISR 2.0, the dominant methanogens influenced by ISR were *Methanosaeta* and *Methanosarcina*. The equations developed in this work can be used and validated in future experiments.

## AUTHOR CONTRIBUTIONS

**Marvin T. Valentin:** Conceptualization (lead); methodology (equal); software (lead); visualization (lead); writing – original draft (lead). **Daniel Ciolkosz:** Conceptualization (equal); review and editing (equal); formal analysis (equal); supervision (equal); validation (equal). **Andrzej Białowiec:** Conceptualization (equal); review and editing (equal); funding acquisition (lead); investigation (equal); validation (equal); visualization (equal).

## CONFLICT OF INTEREST STATEMENT

The authors declare no conflict of interest.

## Supporting information


**Appendix A.** Supporting Information.

**Appendix B.** Supporting Information.

## Data Availability

The data that support the findings of this study are available in the appendix and supplementary material of this article.
