# Supplementary material for: Influence of inoculum‐to‐substrate ratio on biomethane production via anaerobic digestion of biomass
Source: Environ Microbiol Rep. 2024 Dec 2;16(6):e70009. doi: 10.1111/1758-2229.70009 (PMC11610627; doi:10.1111/1758-2229.70009)
Supplement: Supplementary file 1 — Appendix A. Supporting Information. [file EMI4-16-e70009-s002.docx]

APPENDIX A

Figure SA1. The influence of the different levels of ISR on the CH_4_ conversion of various AD experiments from the literature (Demichelis et al., 2022; Moset et al., 2015; Raposo et al., 2009; Rodriguez-chiang and Dahl, 2015).

Table SA1. The maximum methane yield from the anaerobic digestion of various substrates under different inoculum-to-substrate ratios.

| **ISR Values** | **Inoculum** | **Substrate** | **Methane Yield** | | | **Ref** |
| --- | --- | --- | --- | --- | --- | --- |
|  |  |  | Values | Units | Description |  |
| 1 | Sludge | Food waste | 440 | mL-CH_4_ /gVS | Ultimate Methane yield | (Cai et al., 2016) |
| 1 | Municipal Wastewater | Wastewater | 297.4 | mL-CH_4_/gVS | Ultimate Methane yield | (Rodriguez-Chiang and Dahl, 2015) |
| 1 | - | Starch | 370 | mL-CH_4_/gVS | Methane yield | (Raposo et al., 2011) |
| 1 | Brewery wastewater | Sunflower oil | 165 | mL-CH_4_/gVS | Maximum methane generation | (Raposo et al., 2009) |
| 1 | Sludge | Textile waste | 150.92 | mL-CH_4_/gVS | Methane yield | (Juanga-Labayen et al., 2021) |
| 1 | Mixture of livestock manure, poultry manure, wheat grains, whey, potato and tea bags | Simulated food waste | 259 | mL/gVS | Specific methane yield | (Gandhi et al., 2022) |
| 2 | Brewery wastewater | Sunflower oil | 205 | mL-CH_4_/gVS | Maximum methane generation | (Raposo et al., 2009) |
| 2 | Sludge | Textile waste | 336.76 | m-CH_4_/gVS | Methane yield | (Juanga-Labayen et al., 2021) |
| 2 | Mixture of livestock manure, poultry manure, wheat grains, whey, potato and tea bags | Simulated food waste | 323 | mL/gVS | Specific methane yield | (Gandhi et al., 2022) |
| 2 | Municipal Wastewater | Wastewater | 332.9 | mL-CH_4_/gVS | Ultimate Methane yield | (Rodriguez-Chiang and Dahl, 2015) |
| 2 | Sludge | Food waste | 490 | mL-CH_4_ /gVS | Ultimate Methane yield | (Cai et al., 2016) |
| 2 | Rubber latex | Paragrass and pig manure | 437.6 | mL-CH_4_/gTS | Maximum Methane yield | (Dechrugsa et al., 2013) |
| 2 | Pig farm | Paragrass and pig manure | 475.0 | mL-CH_4_/gTS | Maximum Methane yield | (Dechrugsa et al., 2013) |
| 3 | Brewery wastewater | Sunflower oil | 245 | mL- CH_4_/gVS | Maximum methane generation | (Raposo et al., 2009) |
| 3 | Pig farm | Paragrass and pig manure | 519.5 | mL-CH_4_/gTS | Maximum Methane yield | (Dechrugsa et al., 2013) |
| 3 | Rubber latex | Paragrass and pig manure | 465.9 | mL-CH_4_/gTS | Maximum Methane yield | (Dechrugsa et al., 2013) |
| 4 | Pig farm | Paragrass and pig manure | 521.9 | mL-CH_4_/gTS | Maximum Methane yield | (Dechrugsa et al., 2013) |
| 4 | Mixture of livestock manure, poultry manure, wheat grains, whey, potato and tea bags | Simulated food waste | 340 | mL/gVS | Specific methane yield | (Gandhi et al., 2022) |
| 4 | Rubber latex | Paragrass and pig manure | 422.6 | mL-CH_4_/gTS | Maximum Methane yield | (Dechrugsa et al., 2013) |

EQUATION DERIVATION

The working volume of the reactor is comprised of the volume of the inoculum, substrate, and nutrient solution [Figure 1]. Mathematically it is expressed as in Eq. 1. The nutrient solution depends on the requirement or preference of the experiment. It can be disregarded if the substrate being treated does not require nutrients leaving the total working volume with the substrate and inoculum only.

| $V_{w}=V_{subs}+V_{inoc}+V_{ns}$ | (1) |
| --- | --- |


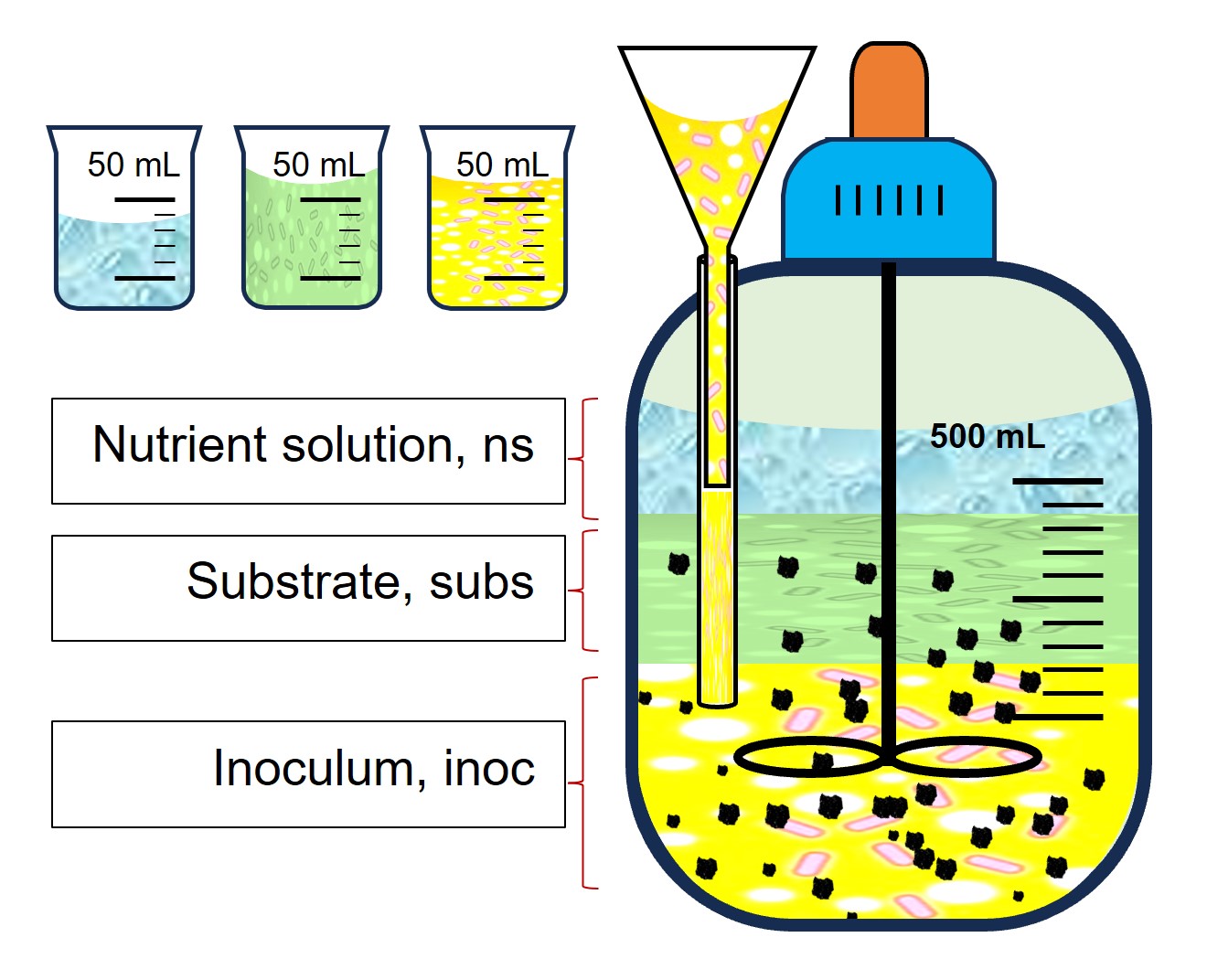


Substrate, subs

Nutrient solution, ns

Inoculum, inoc

Figure 1. Reactor volume composition.

Where $V_{w}$ is the reactive or working volume of the reactor, $V_{subs}$ is the volume of the substrate, $V_{inoc}$ is the volume of the inoculum, and $V_{ns}$ is the volume of the nutrient solution. The working volume is usually a certain percentage of the total reactor volume. Most literature used 80% as a working volume (Rodriguez-chiang and Dahl 2015; Wang et al. 2015; Namal 2020). The remaining 20% is dedicated for the headspace.

To model the change of the volatile solid (VS) of the biomass inside the reactor an ordinary differential equation (ODE) is implemented. The physical law of time rate of change assumes that the change in VS is proportional to the change in time [Eq. 2].

|  | $\frac{dVS}{VS}\propto-kdt$ | (2) |
| --- | --- | --- |

Where $VS$ denotes volatile solids, and $k$ (time^-1^) is the proportionality constant, more appropriately known as the biodegradability factor of the material, assigned with a minus sign to indicate degradation. Applying integration to Eq. 2 results to $\ln[{VS}_{t}]=-kt+c$. Where, ${VS}_{t}$, is the volatile solid at any time $t$ simplified into its exponential form [Eq. 3].

|  | ${VS}_{t}={ce}^{-kt}$ | (3) |
| --- | --- | --- |

In the initial value condition at time zero ($t=0$), the amount of VS present in the reactor is simply its initial quantity (${VS}_{t=0}\underset{\to}{}{VS}_{intl})$ say, initial volatile solid (${VS}_{intl}$). Applying this condition to Eq. 3 gives the value for the constant $c$ as ($c= {VS}_{intl}$) resulting in a particular solution [Eq. 4]. Eq. 3 can be applied in both substrate and inoculum.

|  | ${VS}_{t-s}={({VS}_{intl}) e}^{-k_{s}t}$ | (4) |
| --- | --- | --- |

Where ${VS}_{t-s}$ is the VS of the substrate at any time and $k_{s}$ is the biodegradability constant of the substrate. In the case of inoculum, microbial biomass growth can occur due to the decomposition of VS from the substrate. Considering that microbial biomass growth follows a first-order character, the equation describing the accumulation of microorganisms can have the form [Eq. 5].

|  | ${VS}_{t-i}={VS}_{imax}\left( 1-e^{-k_{i}t} \right)$ | (5) |
| --- | --- | --- |

Where ${VS}_{t-i}$ is the VS of the inoculum at any time, ${VS}_{imax}$ is the maximum biomass growth, and $k_{i}$ is the biodegradability constant of the inoculum. In the preparation of the mixture of substrate and inoculum, their respective quantities are usually based on the volatile solid content of the materials [Eq. 6].

| $ISR=\frac{mass in grams of {VS}_{inoc}}{mass in grams of {VS}_{subs}}$ | (6) |
| --- | --- |

Considering the change in VS of inoculum and substrate over time, Eq. 4 and 5 can be integrated and substituted into Eq. 6 to give Eq. 7.

| $ISR= \frac{{VS}_{imax}\left( 1-e^{-k_{i}t} \right)}{{{VS}_{subs} e}^{-k_{s}t}}$ | (7) |
| --- | --- |

The numerator of Eq. 7, ${VS}_{imax}\left( 1-e^{-k_{i}t} \right)$ represents the VS of the inoculum (${VS}_{inoc}$) and it increases with time. This is for the reason that microorganisms consume the substrate by fermentation. With this, the microorganisms in the inoculum grow rapidly leading to an increase in its volatile solid content due to the principle of biomass growth. However, the biomass growth expressed as grams of VS added cannot be higher than the VS consumed from the substrate. Therefore, the VS of inoculum can be expressed in Eq. 8.

| ${VS}_{inoc}=\left( {VS}_{subs(intl)}-{VS}_{subs(eff)} \right)\left( 1-e^{-k_{i}t} \right)+ {VS}_{inoc(intl)}$ | (8) |
| --- | --- |
| ${VS}_{inoc}= {VS}_{u}\left( 1-e^{-k_{i}t} \right)+ {VS}_{inoc(intl)}$ | (9) |

In Eq. 8, the difference in the initial VS of the substrate (${VS}_{subs(intl)}$) and final (effluent) VS of the substrate (${VS}_{subs(eff)}$) is the amount of VS solids available for the microorganisms. In other studies, this is referred to as the ultimate VS degraded at the time, $t$ (Kavitha et al. 2016). For simplicity, this difference is renamed as, ${VS}_{u}$ [Eq. 9]. Biomass growth from this scenario will be in the order $1-e^{-k_{i}t}$. Adding this to the initial VS of the inoculum (${VS}_{inoc(intl)}$) can give the VS of the inoculum at any time. The final VS of the substrate can be calculated using Eq. 4 considering the half-life principle ($t_{1/2}=ln2/k_{s}$). With this, Eq. 9 can be integrated into Eq. 4 to give Eq. 10.

| $ISR= \frac{{VS}_{u}\left( 1-e^{-k_{i}t} \right)+{VS}_{inoc(intl)}}{{{VS}_{subs} e}^{{-k}_{s}t}}$ | (10) |
| --- | --- |

The mixture is more convenient to prepare in terms of the respective initial masses of the materials. As such, Eq. 10 is expressed in terms of mass [Eq. 11] where, ${FM}_{i}$, refers to the fresh mass of the inoculum, ${FM}_{subs}$, refers to the fresh mass of the substrate, ${DM}_{inoc}$ and ${VS}_{inoc}$, are the percent dry mass and percent volatile solid of the inoculum in decimal, respectively, while, ${DM}_{subs}$, and ${VS}_{subs}$, represent the percent dry mass and percent volatile solid of the substrate in decimal.

| $ISR= \frac{{VS}_{u} \left( 1-e^{-k_{i}t} \right)+{FM}_{inoc}{DM}_{inoc}V_{inoc}}{{FM}_{subs}{DM}_{subs}{{VS}_{subs} e}^{-k_{s}t}}$ | (11) |
| --- | --- |

Furthermore, the ISR can be expressed in terms of the corresponding volume of inoculum and substrate [Eq. 12], where $V_{inoc}$ and $V_{subs}$ are the volumes of inoculum and substrate, respectively, and, $\rho_{inoc}$ and $\rho_{subs}$ are the respective bulk densities of the inoculum and substrate. This would be convenient to use especially if the substrate is in liquid form. However, in most cases, the substrate comes in solid form while the inoculum is liquid. With this, the denominator of Eq. 11 is written in terms of fresh mass.

| $ISR=\frac{{VS}_{u}\left( 1-e^{-k_{i}t} \right)+{\rho_{inoc} V_{inoc}DM}_{inoc}{VS}_{inoc}}{{\rho_{subs} V_{subs}DM}_{subs}{{VS}_{subs} e}^{-k_{s}t}}$ | (12) |
| --- | --- |
